# Irisin Protects Against Motor Dysfunction of Rats with Spinal Cord Injury via Adenosine 5'-Monophosphate (AMP)-Activated Protein Kinase-Nuclear Factor Kappa-B Pathway

**DOI:** 10.3389/fphar.2020.582484

**Published:** 2020-11-16

**Authors:** Xi Jiang, Zhihong Shen, Jin Chen, Chao Wang, Zhan Gao, Songling Yu, Xuefeng Yu, Lei Chen, Lexing Xu, Ziwei Chen, Wenjuan Ni

**Affiliations:** ^1^ Zhejiang University Mingzhou Hospital, Ningbo, China; ^2^ Department of Pharmacy, Zhejiang Pharmaceutical College, Ningbo, China; ^3^ Ningbo Yinzhou No.2 Hospital, Ningbo, China

**Keywords:** irisin, spinal cord injury, motor function, lipopolysaccharide, adenosine 5'-monophosphate (AMP)-activated protein kinase, nuclear factor kappa-B

## Abstract

The aim of the present research was to investigate the effects of irisin, a skeletal muscle-derived myokine, on spinal cord injury (SCI) in rats and explore the possible mechanisms. SCI model was constructed in male SD rats. The effects of irisin on SCI rats were assessed via behavior tests including Basso, Beattie, and Bresnahan (BBB) scoring method and inclined plane test, followed by histomorphology tests including HE staining, Nissl staining, and transmission electron microscope examination. Biochemical analyses including PCR, Western blots and ELISA were employed to further evaluate the changes at molecular level of SCI rats. In addition, lipopolysaccharide (LPS)-induced cell damage model was established in PC12 cells to verify the mechanism of irisin’s effect on nerve cells *in vitro*. Results showed that the BBB score and the angle of incline significantly decreased after SCI surgery, however, chronic irisin treatment improved SCI-induced motor dysfunction. HE and Nissl staining assays showed that SCI surgery induced histological injury of spinal cord, which could be reversed by irisin treatment. Morphological abnormality of nerve cells caused by SCI also could be alleviated by irisin. Further biochemical analyses showed that irisin inhibited SCI-induced overexpression of Interleukin-1β (IL-1β), Interleukin- 6 (IL-6), tumor necrosis factor alpha (TNF-α), inducible nitricoxidesynthase (iNOS) and Cyclooxygenase-2 (COX-2)], as well as nuclear factor kappa-B (NF-κB)p65 in rats, and the positive function of irisin could be reversed by Compound C treatment. In our *in vitro* study, LPS-induced declines of cell viability and neurite length of PC12 cell were inhibited by irisin treatment, and irisin inhibited LPS-induced overexpression of NF-κBp65, IL-1β, IL-6, TNF-α, iNOS and COX-2. These changes could be reversed by activated protein kinase (AMPK) siRNA pre-treatment. Taken together, irisin could protect the rats from SCI, and its protection is associated with the regulation of **adenosine 5'-monophosphate-activated protein kinase (AMPK)**- NF-κB signaling pathway.

## Introduction

Spinal cord injury (SCI) is one of the devastating traumas in central nervous system diseases, which leads to paralysis, palsy and even death in severe cases (Singh et al., 2014). Reports spanning various countries demonstrated that SCI has become a common disease affecting millions of individuals worldwide (Noonan et al., 2012; Smith et al., 2010–2015; Saberi et al., 2016). Though great efforts have been made to seek effective treatment method for SCI (Ma et al., 2020; Zheng et al., 2020), no optimal therapeutic strategy has been achieved yet. Thus, continuing efforts of exploring effective and safe drug to treat SCI are needed.

Irisin is a skeletal muscle-derived myokine. It was firstly known as a product of Fibronectin type III domain-containing protein 5, a protein that is increased with exercise in mice and humans (Bostrom et al., 2012). The subsequently induced irisin in muscle and blood can cause an increase in energy expenditure. Therefore, irisin was deemed to be therapeutic for human metabolic disease such as diabetes and obesity (Huh et al., 2012; Peterson et al., 2014; Crujeiras et al., 2015). As physical exercise possesses numerous benefits, the exercise-induced hormone irisin has attracted considerable attention. In the past few years, irisin was demonstrated to have protective effects on intestine, bowel, heart and liver (Narayanan et al., 2018; Kheiripour et al., 2019; Ren et al., 2019; Zhao et al., 2019). Moreover, some studies have shown that irisin is beneficial to nerve cells (Kim and Song, 2018). However, these studies mainly focused on brain, rarely on spinal cord. The function of irisin demonstrated by the previous researches mainly depends on **adenosine 5'-monophosphate-activated protein kinase** (AMPK) activation mechanism. A previous study demonstrated irisin could protect against endothelial injury and ameliorated atherosclerosis in apoE (−/−) diabetic mice through activation of AMPK-PI3K-Akt-eNOS signaling pathway (Lu et al., 2015). Another study suggested that irisin facilitates glucose and lipid metabolism in human muscle through AMP kinase phosphorylation (Huh et al., 2014). Liu and his colleagues found that irisin could inhibit pancreatic cancer by activating AMPK pathway and suppressing the mammalian target of rapamycin signaling (Liu et al., 2018). These studies revealed the strong correlation between AMPK and the function of irisin. (Jiang et al., 2019). SCI process includes primary injury referring to mechanical compression to spinal cord, nerve roots and osseous structures at initial impact, and secondary injury resulting from subsequent neuroinflammation and oxidative stress. As is known, AMPK is a critical kinase participated in energy metabolism and a regulator of nuclear factor-kappaB (NF-κB), and neuroinflammation and oxidative stress are two vital pathogenesis involved in various diseases that are regulated by NF-κB (Csaki et al., 2009; Wang et al., 2019), it seems that irisin has some correlation with SCI. Here we tried to investigate the possible role of irisin in the remission of SCI in perspective of the secondary injury.

In this study, we determined the function of irisin on SCI via assessing the locomotor activity and spinal cord histomorphology of SCI rats after irisin treatment. The changes of AMPK and its downstream factors including NF-κB, IL-1β, IL-6, TNF-α, Cyclooxygenase-2 (COX-2) and inducible nitricoxidesynthase (iNOS) in animal tissues and PC 12 cells were assessed to investigate the possible mechanism. Oxidative-stress related factors Mn-superoxide dismutase (MnSOD), glutathione (GSH) and malondialdehyde (MDA) in PC 12 cells were also determined to preliminary explore whether irisin’ function was associated with antioxidant effect.

## Materials and Methods

### Animals

Adult Male SD rats (220–240 g) purchased from Shanghai Experimental Animal Center, Chinese Academy of Sciences were used in this study. The rats were housed five per cage under controlled environmental condition. Prior to experiment, the animals were allowed to adapt to the environment for one week. A total of 72 rats were equally allocated into six groups (n = 12 per group): sham (animals underwent surgery without SCI opera-tion), sham + irisin (10 *μ*g/kg, i.v.), SCI, SCI + irisin (5 *μ*g/kg, i.v.), SCI + irisin (10 *μ*g/kg, i.v.), SCI + irisin (10 *μ*g/kg, i.v.) + compound C (AMPK inhibitor) (10 mg/kg, i.p.). All the experimental procedures were approved by the Animal Care and Use Committee of Zhejiang Pharmaceutical College (ethical clearance number: wydw2018-0113), and conducted in accordance with the guidelines set by the Chinese National Institutes of Health.

### Treatment Schedule

**FIGURE 1:**
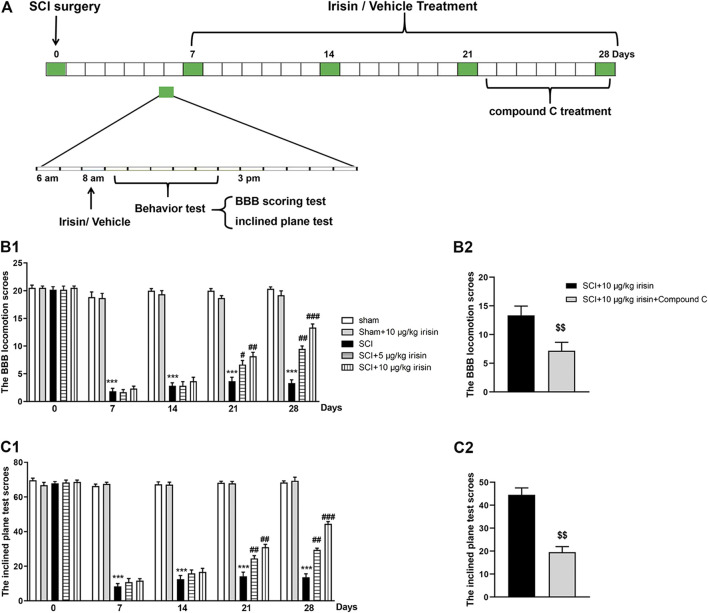
**(A)** Experimental design. Spinal cord compression injury model was chosen to establish spinal cord injury (SCI) rat model. In surgery, rats were performed moderate compression injuries by using a vascular clip for 2 min, sham group received the same surgical procedures without vascular clip. After SCI surgery, rats received administrations (8:00 AM) of irisin (5, 10 *μ*g/kg, i.v.) or vehicle, and Basso, Beattie, and Bresnahan (BBB) scoring method and inclined plane test were used to assess the rats’s motor function on day 0, 7, 14, and 28 post-surgery. Animals were sacrificed on day 28 after behavior tests for neurochemical analysis. **(B1,B2)** data of BBB locomotion test. BBB score was significantly decreased after SCI surgery (*p* = 0.000007 for day 7). The decrease was strongly suppressed by 10 *μ*g/kg irisin (*p* = 0.00012 for day 28). The function of irisin could be reversed by Compound C (*p* = 0.0075). **(C1,C2)** data of inclined plane test. Inclined plane test score was significantly decreased after SCI surgery (*p* = 0.000002 for day7). Irisin treatment suppressed this decrease (*p* = 0.00043 for day 28 in 10 *μ*g/kg irisin group), and this phenomenon was reversed by Compound C (*p* = 0.0032). Data are presented as Mean ± SEM, n = 8. ***p < 0.001 when compared with sham group; ^#^ *p* < 0.05, ^##^ *p* < 0.01 and ^###^ *p* < 0.001 when compared with SCI group; ^$$^ *p* < 0.01 when compared with irisin-treated SCI group.

All the rats underwent SCI surgery except sham and sham + irisin groups. After recovered for 7 days post-surgery, the rats in irisin-treatment groups received irisin (5 or 10 *μ*g/kg, i.v.) for 28 consecutive days (each day at 8 am). Doses of irisin were selected based on previous studies (Zhao et al., 2018; Aydogdu et al., 2019). Irisin solutions were prepared via dissolving irisin powder (a recombinant protein of animal origin that can be used in human, rat, mouse and canine experiments, catalogue number: 067-29A, Phoenix Pharmaceuticals Inc., Burlingame, USA) in normal saline. In sham and SCI groups, equal volume of vehicle was intravenously administrated into rats instead of irisin. Rats in compound C-treatment group received compound C (Cell Signaling Technology, Inc., Shanghai, China) (10 mg/kg, i.p.) during the last seven days. Dose of compound C was chosen based on our previous study (Jiang et al., 2019). Each animal received behavioral test on day 0, 7, 14, 21, and 28 post-surgery. On day 28, blood samples were collected from the tail of rats before sacrifice for the detection of serum irisin level. After anesthesia by pentobarbital (4 mg per 100 g body weight, i.p.), all the animals were decapitated, spinal cord (from T8 toT10) were collected. Plasma was used for testing irisin level by ELISA, and spinal cord tissues were used for staining, transmission electron microscopic examination, PCR, western blot and ELISA. The experimental design is summarized in Figure 1A.

### Spinal Cord Injury Surgery

SCI surgery was carried out as previously described (Zhao et al., 2016). The rats were first anaesthetized by pentobarbital (4 mg per 100 g body weight, i.p.), and then placed in a prone position on a platform. After fixing the animal and exposing level T9 of the spinal cord, a vascular clip of T9 spinal cord for 2 min (30 g forces, Oscar, China) was conducted to induce spinal cord injury. Rats in the sham group underwent the same surgical procedure without clip compression. After that, muscle and skin were sutured with 3–0 vicryl sutures. Postoperative care included bladder massage twice a day for three days and passive mobilization of hind legs three times a day for 28 days.

### Locomotor Function Assessment

Locomotor function of rats was evaluated by Basso, Beattie, and Bresnahan (BBB) scoring test (Basso et al., 1995) and inclined plane test (Dinh et al., 2009) on day 0, 7, 14, 21 and 28 after SCI surgery. In BBB scoring test, activity of hind limb of each animal was videotaped and recorded by three blinded observers. BBB scores contains 21 points, representing 21 grades from no hind limb movement (grade 0) to normal gait (grade 21). In the inclined plane test, the value of the maximum angle at which the animal could maintain for 5 s without falling was recorded as the data of this test.

### HE staining and Nissl Staining

Rats were anesthetized by pentobarbital (4 mg per 100 g body weight, i.p.) and irrigated with physiological saline, followed by formaldehyde PBS solution. The spinal cord tissue (from T8 to T10) was then retrieved and embedded in paraffin. The lesion epicenter was stained with haematoxylin and eosin (HE staining) or cresyl violet (Nissl staining) following the standard protocols (HE Staining Kit and Nissl Staining Kit, purchased from Solarbio Science and Technology Co., Ltd. Beijing). All stained sections were further examined under a light microscope (Nikon, Japan).

### Transmission Electron Microscopic Examination

Rats were anesthetized by pentobarbital (4 mg per 100 g body weight, i.p.) and irrigated with physiological saline, followed by formaldehyde PBS solution. Spinal cord tissue (from T8 to T10) were dissected out and bathed in 2.5% glutaraldehyde for 2 h. After being dehydrated and washed, samples were post-fixed in 1% osmium tetroxide containing 0.8% potassium ferrocyanide and 0.1 M cacodylate buffer including 5 nM calcium chloride for 1.5 h. Samples were then dehydrated in graded acetone, infiltrated with Poly/Bed 812 resin (Polysciences, Inc., Washington, PA) and polymerized for 60 h. Five hundred-nanometre-thick sections were cut on an ultramicrotome (Leica ultracut UCT) and stained with toluidine blue. Images were acquired with a digital camera (DP 11, Japan) attached to a microscope (Olympus Ax70).

### Measurement of Cell Viability and Neurite Length

PC12 cells purchased from the Cell Storage Center of Wuhan University (catalogue number: GDC006, Wuhan, China) were seeded on 96-well plates (5 × 10^3^ cells/well) and cultured in DMEM culture medium supplemented with heat-inactivated 10% fetal bovine serum, 5% horse serum, and antibiotics (100 units/ml penicillin and 100 *μ*g/ml streptomycin) under a humidified atmosphere containing 5% CO_2_ at 37 °C for 24 h. Then, cells were treated with different concentrations of lipopolysaccharide (LPS: 0.1, 0.5, 1, 5 and 10 *μ*g/ml) for 12 h. Afterwards, cells were incubated in medium with 10 *μ*l CCK-8 solution (Abcam, Shanghai, China) for 1 h. Absorbance was measured at 450 nm wavelength with a reference wavelength of 650 nm by a spectrophotometer (Multiskan Spectrum, Thermo Scientific). Data of cell viability was calculated from the value of optical density.

To determine the effect of irisin on LPS-induced injury of PC12 cells, PC12 cells were cultured with irisin (doses of 10, 20, 40 nmol/L) for 12 h following a 12-h LPS (5 *μ*g/ml) treatment. Doses of irisin were selected based on the previous study (Peng et al., 2017). The cell survival rate was then determined. The morphology of viable cell was captured and neurite length was measured using ImageJ software (NIH, Baltimore, MD).

To explore the role of AMPK in the process of nerve cell injury, transfection with siRNAs for AMPK gene silencing in cells was conducted. siRNA oligonucleotides (20 nM) specific for AMPK were purchased from Santa Cruz Biotechnology. To suppress gene expression, cell transfection was performed with Lipofectamine 2000 (Invitrogen) according to the manufacturer’s instructions. In brief, the cells were transfected with validated siRNA or scramble siRNA at a final concentration of 20 nM in the presence of transfection reagent. After transfection, the cells were harvested to use in LPS challenging experiment.

### Measurement of Oxidative-Stress Markers

MnSOD activities were measured by the cytochrome c reduction method as reported previously (Wani et al., 2014). Mitochondrial GSH was estimated by the method mentioned in a previous study (Kaur et al., 2007). Briefly, an equal volume of 1% (w/v) sulfosalicylic acid was added to an aliquot of mitochondrial fraction, then the sample was mixed and centrifuged at 10,000 g for 10 min. The supernatant was collected and 0.4 M Tris buffer (pH 8.9) and 0.1 ml of 0.01 M 5,50-dithiobis-(2-nitrobenzoic acid) (DTNB) was added. The absorbance was then measured at 412 nm. The results were expressed as nmol GSH/mg protein. MDA expression was assessed according to the previously described method (Xu et al., 2013).

### Quantitative Real-Time PCR

mRNA levels of iNOS and COX-2 in spinal cord were detected by PCR. Tissue samples were prepared according to the RNA kit (Bio-Rad. Labs). Total cellular RNA was isolated using Trizol reagent (Trizol Invitrogen). RNA concentration was determined using a spectrophotometer (Bio-Rad. Labs) at 260 nm. The PCR reaction was performed using iCycler Real-Time PCR machine (Bio-Rad, Hercules CA, USA). SYBR Green (iQ SYBR Green supermix reagent, Bio-Rad) was added to each sample at a concentration of 50 nmol/L. Protocol of the real-time PCR was as follows: initial denaturation at 95°C for 10 min, followed by 40 cycles at 95°C for 10 s, 58°C for 30 s. At the end of the PCR reaction, a melting curve was obtained by holding at 95°C for 15 s, cooling to 60°C for 1 min, and then heating slowly at 0.5°C/s until 95°C. The primer sequences of target RNAs were shown in Table 1. All the data were normalized to the housekeeping gene, β-actin.

**TABLE1:** The primer sequences of iNOS and COX-2.

Target	Forward (5′-3′)	Reverse (5′-3′)
iNOS	CCTCCTCCACCCTACCAAGT	CACCCAAAGTGCTTCAGTCA
COX-2	TGGGTGTGAAAGGAAATAAGGA	GAAGTGCTGGGCAAAGAATG
β-actin	TGGAATCCTGTGGCATCCATGAAAC	AAAACGCAGCTCAGTAACAGTCCG

### Western-Blot Analysis

Protein levels of AMPK and pAMPK in spinal cord tissue and PC 12 cells were detected by Western-blot analysis. BCA kit (Thermo Scientific) was used to determine protein concentration of each sample. Each band contains a total protein of 40 *μ*g. After electrophoresis and membrane transferring, blots were blocked with milk for 2 h and then incubated with primary antibodies (anti-pAMPK 1:1,000 and anti-AMPK 1:1,000, purchased from abcam; anti-β-actin 1:1,000, purchased from santa cruz). After being washed and incubated with secondary antibodies, blots were imaged by fluorescence scanner (LI-COR Biotechnology, South San Francisco, CA, USA) and data were analyzed.

### ELISA

Protein levels of IL-1β, IL-6, TNF-α and NF-κBp65 in spinal cord tissues and PC 12 cells were detected by ELISA kit purchased from Thermo Scientific (Shanghai, China). Protein levels of iNOS and COX-2 in spinal cord tissues and PC 12 cells were detected using the ELISA Detection Kits purchased from Abcam (Shanghai, China). The OD values of iNOS, COX-2, IL-1β, IL-6 and TNF-α in each sample were detected at 450 nm wavelength. Only the OD value of NF-κBp65 was measured at 405 nm wavelength.

Irisin levels in the spinal cord and serum were measured with ELISA kit (Phoenix Pharmaceuticals, Burlingame, CA, USA). The lowest detectable concentration of irisin was 1.29 ng/ml (Aydin et al., 2017). Sample absorbance was read at 450 nm wavelength with an ELX 800 ELISA reader.

### Statistical Analysis

A total of 72 rats (n = 12 in each group) were used for data analysis. In each group, all the rats were assessed for behavior performance, four rats were used for staining assays, two rats were used for transmission electron microscopic examination, and six rats were used for PCR, Western-blot and ELISA experiments. Multiple-group comparisons were analyzed by one-way ANOVA via SPSS software (International Business Machines Corporation, IBM, USA). Two group comparisons were analyzed by dunnett test. Results were expressed as mean ± SEM, with significance taken as value of *p* < 0.05.

## Results

### Effects of Exogenous Irisin on Spinal Cord Injury-Induced Motor Dysfunction in Rats

As shown in Figure 1B, SCI surgery induced a significant decrease of BBB score (*p* < 0.001). However, chronic treatment with irisin (10 *μ*g/kg, i.v.) increased the BBB score in rats underwent SCI surgery. On day 28, the maximal improving effect was achieved in SCI + 10 *μ*g/kg irisin group (*p* < 0.001, Figure 1B1), while Compound C treatment inhibited the improving effect of irisin (*p* < 0.01, Figure 1B2).

Similarly, the angle of incline was significantly decreased after SCI surgery (*p* < 0.001) (Figure 1C1), and treatment with irisin (10 *μ*g/kg, i.v.) ameliorated this adverse effect (*p* < 0.01). Interestingly, the effects of irisin on SCI rats’ motor function were reversed by compound C treatment (*p* < 0.001, Figure 1C2) on day 28.

### Spinal Cord Histology of Rats

**FIGURE 2:**
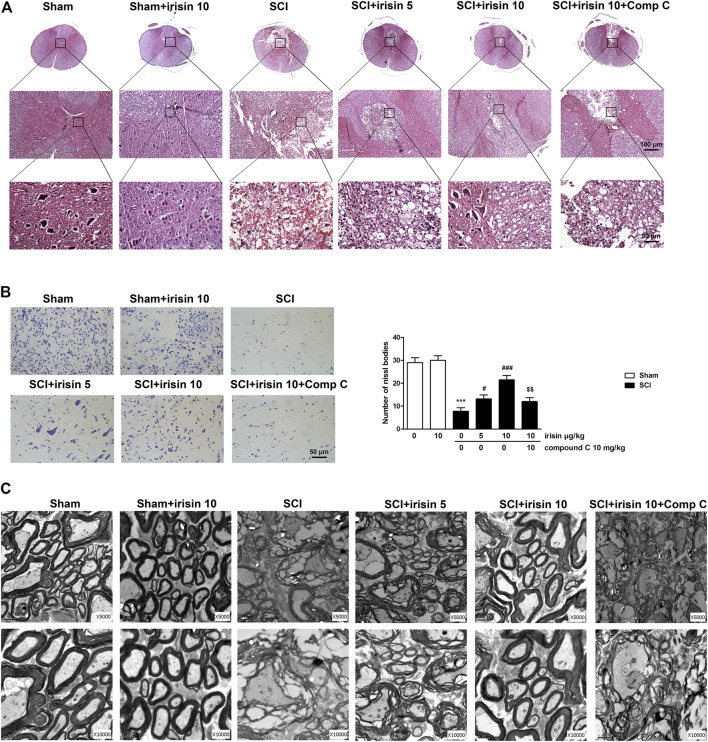
**(A)** HE staining on transverse section of spinal cord at T9 in sham and spinal cord injury (SCI) rats at the 28th day after spinal cord injury (The first row is ×50, second is ×200 and third is ×400); **(B)** Nissl staining (×40) on transverse section at T9 in rats of sham and SCI, and quantitative analysis of Nissl bodies in staining images (n = 4), scale bar = 50 *μ*m. SCI surgery decreased Nissl bodies on day 28 (*p* = 0.00009), and irisin treatment improved this decrease (*p* = 0.00021 in 10 *μ*g/kg irisin group). Irisin’s function were reversed by compound C treatment (*p* = 0.0039). **(C)** Ultrastructural morphology of myelin sheath and neuronal cells in the dorsal column and epicenter surrounding gray matter of different groups. Micrographs represent samples taken from two rats in each group. Data are presented as Mean ± SEM. ***p < 0.001 when compared with sham group; ^#^ *p* < 0.05 and ^###^ *p* < 0.001 when compared with SCI group; ^$$^ *p* < 0.01 when compared with irisin-treated SCI group. Comp C: Compound C.

Results of HE staining for spinal cord samples were shown in Figure 2A. Morphology of normal spinal cord neurons was characterized by clear cell contour and cytoplasm with uniform nuclei as observed in sham groups. However, after SCI surgery, lesion center characterized by the destruction of gray and white matter could be easily found. The neurons in the anterior horn shrunk or had pale homogenous cytoplasm. Tissue repairment in spinal gray matter was observed after several times of irisin treatment, including the recovery of nuclei and morphology, and reduction of organization interspace. Better protective effect of irisin on spinal cord injury could be seen in SCI + 10 *μ*g/kg irisin group as compared with SCI + 5 *μ*g/kg irisin group. While the protective effect of irisin on spinal cord neurons was partially abolished by compound C.

Similar protective effect of irisin on SCI rats were observed in the results of Nissl staining. In rats undergoing SCI surgery, Nissl bodies in the anterior horns were significantly decreased when compared with the sham group (*p* < 0.001, Figure 2B), however, irisin treatment reversed SCI-induced decreasing of Nissl bodies (*p* < 0.001). Interestingly, the effects of irisin on spinal cord injury shown in Nissl staining assay were suppressed by compound C (*p* < 0.01).

### Spinal Cord Neurons Morphology of Rats

To further confirm the improving effect of irisin on spinal cord injury, ultrastructural analysis of the epicenter and its surrounding area was performed on day 28 (Figure 2C). In the sham group, nerve cells showed normal morphology, and the axons were myelinated with a compact multilayered sheath. SCI surgery induced obvious cellular damage, including dissolved cavitation, karyopyknosis and degenerated myelin sheath with a loose state. However, irisin treatment reversed these phenomena, especially 10 *μ*g/kg irisin treatment. Nevertheless, compound C treatment impeded irisin’s improving effect on morphology of spinal nerve cells in SCI rats.

### Irisin Levels in Serum and Spinal Cord of Rats

**FIGURE 3:**
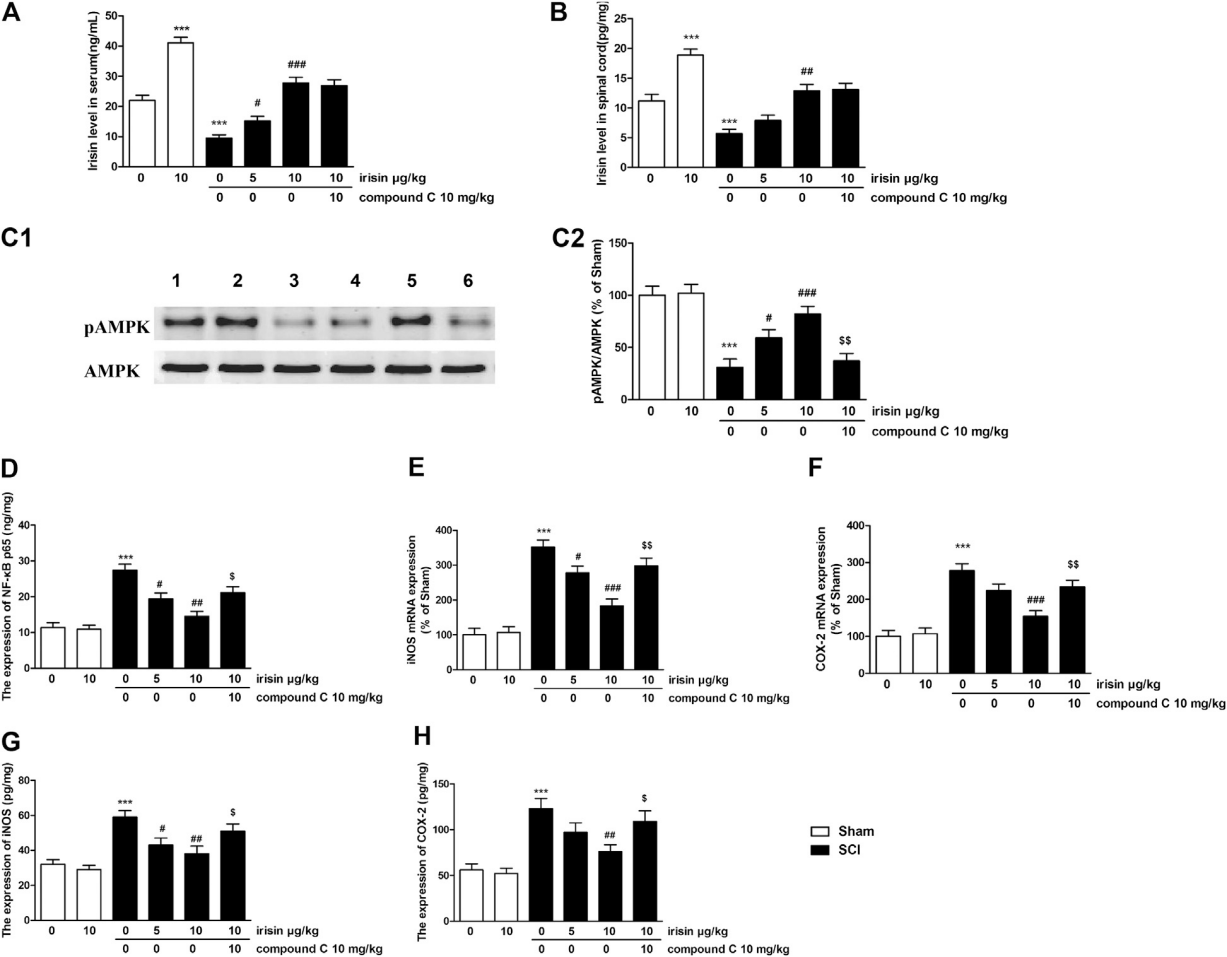
**(A)** Expression of irisin in the serum of sham/spinal cord injury (SCI) rats (***p = 0.00011 for SCI group, ^###^ *p* = 0.00008 for SCI + 10 *μ*g/kg irisin group). **(B)** Expression of irisin in the spinal cord of sham/SCI rats (***p = 0.00018 for SCI group, ^###^ *p* = 0.0017 for SCI + 10 *μ*g/kg irisin group). **(C)** Effects of irisin on pAMPK expression in the spinal cord in sham/SCI rats, and the quantitative analysis of the blots (***p = 0.00015, ^###^ *p* = 0.00078, ^$$^ *p* = 0.0014). blot 1: sham; 2: irisin-treated sham; 3: SCI; 4–5: irisin (5, 10 *μ*g/kg, i.v) treated SCI; 6: compound C treated group. **(D)** Effects of irisin on NF-κBp65 expression in the spinal cord in sham/SCI rats (***p = 0.000027, ^##^ *p* = 0.0016, ^$^ *p* = 0.0134). **(E,F)** Effects of irisin on the mRNA expression of inducible nitricoxidesynthase (iNOS) (***p = 0.000012, ^###^ *p* = 0.00013, ^$$^ *p* = 0.0034) and COX-2 (***p = 0.000032, ^###^ *p* = 0.00047, ^$$^ *p* = 0.007) in the spinal cord of sham/SCI rats. **(G,H)** Effects of irisin on expressions of iNOS (***p = 0.00017, ^##^ *p* = 0.0051, ^$^ *p* = 0.047) and Cyclooxygenase-2 (***p = 0.00044, ^##^ *p* = 0.0058, ^$^ *p* = 0.038) in the spinal cord of sham/SCI rats. Data are presented as Mean ± SEM, n = 6. ***p < 0.001 when compared with sham group; ^#^ *p* < 0.05, ^##^ *p* < 0.01 and ^###^ *p* < 0.001 when compared with SCI model group; ^$^ *p* < 0.05 and ^$$^ *p* < 0.01 when compared with irisin-treated SCI group.

Irisin levels in serum and spinal cord were detected on day 28 after SCI surgery. As shown in Figures 3A,B, SCI surgery significantly decreased irisin levels in serum and spinal cord (*p* < 0.001 for serum, Figure 3A; *p* < 0.001 for spinal cord, Figure 3B), when compared with the sham rats. Exogenous irisin treatment increased irisin levels in the serum and spinal cord. The variation trends of irisin levels in different groups in serum and spinal cord were consistent.

### Effects of Exogenous Irisin on Spinal Cord Injury-Induced Changes of AMPK Signaling Pathway in Rats

Data of AMPK expressions were summarized in Figure 3C. Significant decrease of pAMPK expression in the spinal cord (*p* < 0.001) was observed 28 days after SCI surgery. Irisin (10 *μ*g/kg) treatment significantly reversed these reductions (*p* < 0.001). For NF-κBp65, overexpression of NF-κBp65 was found in the spinal cord of SCI rats (*p* < 0.001, Figure 3D), while 10 *μ*g/kg irisin treatment greatly inhibited the expressions of NF-κBp65 (*p* < 0.01). Pro-inflammatory cytokines (IL-1β, IL-6, TNF-α), the response elements of NF-κBp65, were dramatically increased in the spinal cord (*p* < 0.01 for TNF-α, *p* < 0.001 for IL-6 and IL-1β, Table 2). These increases were inhibited by chronic irisin (10 *μ*g/kg) treatment. Furthermore, SCI surgery induced overexpression of iNOS and COX-2 mRNA in the spinal cord (*p* < 0.001 for iNOS and COX-2, Figures 3E,F). Similarly, increments were also observed in the ELISA data of iNOS and COX-2 (*p* < 0.001 for iNOS and COX-2, Figures 3G,H). Interestingly, the effects of irisin on expressions of AMPK, NF-κBp65, IL-1β, IL-6 TNF-α iNOS and COX-2 were suppressed by compound C.

**TABLE 2:** Effects of irisin on IL-1β, IL-6 and TNF-α expressions of spinal cord in rats.

Group	Irisin dose (*μ*g/kg)	Spinal cord (pg/mg per tissue)		
		IL-1β	IL-6	TNF-α
Sham	0	7.5 ± 1.4	6.2 ± 1.5	7.0 ± 1.7
Sham + irisin	10	7.3 ± 1.6	6.4 ± 1.3	6.8 ± 1.6
SCI	0	18.4 ± 1.7^***^	15.2 ± 1.9^***^	12.5 ± 2.1^**^
SCI + Irisin	5	14.1 ± 1.9	9.0 ± 1.7^#^	10.2 ± 1.5
Comp C + irisin + SCI	10	8.5 ± 1.6^##^	7.4 ± 1.5^##^	7.9 ± 1.2^##^
	10	16.9 ± 1.4^$$^	12.5 ± 1.8^$^	11.7 ± 1.6^$^

Comp C, Compound C; SCI, spinal cord injury. Values were expressed as mean ± SEM. Each group contains six rats. Data analysis was performed using Dunnett’s t-test. ^**^ *p* < 0.01 and ^***^ *p* < 0.001 vs. sham group; ^#^ *p* < 0.05 and ^##^ *p* < 0.01 vs. SCI group; ^$^ *p* < 0.05 and ^$$^ *p* < 0.01 vs. SCI + 10 *μ*g/kg irisin group.

### Effects of Irisin on Lipopolysaccharide-Induced Cell Survival Rate Decline and Neurite Growth Inhibition in PC12 Cells

**FIGURE 4:**
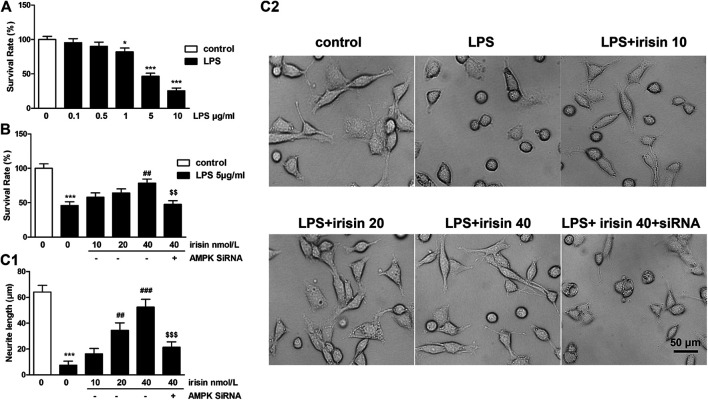
**(A)** Survival rates of PC12 cells challenged with different doses of lipopolysaccharide (LPS) (*p* = 0.000015 for 5 *μ*g/ml LPS group). **(B)** Effects of irisin (dose of 10, 20, 40 nmol/L) on the survival rate of PC12 cells challenged with 5 *μ*g/ml LPS (***p = 0.000072, ^##^ *p* = 0.0022, ^$$^ *p* = 0.003). **(C1-2)** Neurite length of P12 cells treated with irisin followed by 5 *μ*g/ml LPS pretreatment (***p = 0.000031, ^###^ *p* = 0.000052, ^$$$^ *p* = 0.00093). Scale bar = 50 *μ*m. Data are presented as Mean ± SEM, n = 5. *p < 0.05, **p < 0.01 and ***p < 0.001 when compared with control without LPS group; ^#^ *p* < 0.05, ^##^ *p* < 0.01 and ^###^ *p* < 0.001 when compared with LPS group; ^$$^ *p* < 0.01 and ^$$$^ *p* < 0.001 when compared with irisin-treated LPS group.

Firstly, an optimal PC12 cellular injury model was established by challenging PC 12 cells with different concentrations of LPS. The cell viability reached 46.7 ± 4.3% when 5 *μ*g/ml LPS was used, while all the cells almost died when 10 *μ*g/ml LPS was used (Figure 4A). Therefore, the concentration of 5 *μ*g/ml of LPS was selected in further cell experiment. Furthermore, PC 12 cells were treated with irisin (10, 20, 40 nM) in basal condition to determine whether irisin affect the viability of PC 12 cells. Data indicated that irisin had no effect on PC12 cell survival.

The result of cell viability assay showed that treatment of 5 *μ*g/ml LPS resulted in a cell survival rate of 45%, whereas pretreatment with 40 nmol/L of irisin significantly attenuated LPS-induced cell damage (*p* < 0.01, Figure 4B). PC12 cells with AMPK gene silencing did not response to irisin’s protective function.

For neurite length, 5 *μ*g/ml of LPS significantly decreased neurite length of PC12 cells (*p* < 0.01, Figure 4C1-2), and neurite length of irisin-treated (40 nmol/L) cells was 52.6 ± 5.9 *μ*m, which was dramatically longer than that of injured cells (7.5 ± 3.2 *μ*m). Furthermore, pretreatment with AMPK siRNA reversed irisin’s function on neurite growth.

### Effects of Irisin on Lipopolysaccharide-Induced Changes of AMPK Signaling Pathway in PC12 Cells

**FIGURE 5:**
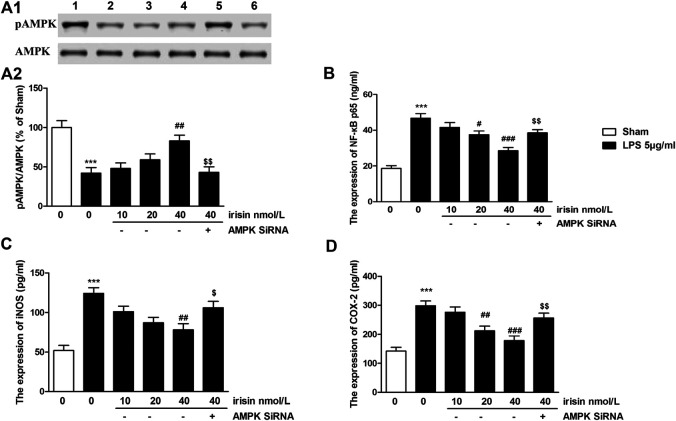
**(A)** Effects of irisin on pAMPK expression in lipopolysaccharide (LPS)-challenged PC12 cells, and the quantitative analysis of the blots (***p = 0.00044, ^##^ *p* = 0.0025, ^$$^ *p* = 0.0029). blot 1: control; 2: LPS; 3–5: irisin (doses of 10, 20 and 40 nmol/L) + LPS; 6: compound C + irisin (40 nmol/L) + LPS group. **(B)** Effects of irisin on NF-κBp65 expression in in LPS-treated PC 12 cells (***p = 0.000011, ^###^ *p* = 0.00021, ^$$^ *p* = 0.0026). **(C,D)** Effects of irisin on expressions of inducible nitricoxidesynthase (iNOS) (^***^ *p* = 0.000019, ^##^ *p* = 0.0015, ^$^ *p* = 0.037) and Cyclooxygenase-2 (***p = 0.000026, ^###^ *p* = 0.00043, ^$$^ *p* = 0.0074) in LPS-treated PC 12 cells. Data are presented as Mean ± SEM, n = 5. ***p < 0.001 when compared with control group; ^#^ *p* < 0.05, ^##^ *p* < 0.01 and ^###^ *p* < 0.001 when compared with LPS group; ^$^ *p* < 0.05 and ^$$^ *p* < 0.01 when compared with irisin-treated LPS group.

The expression of AMPK in PC12 cells were presented in Figure 5A. 5 *μ*g/ml of LPS induced significant decreases of pAMPK expression (*p* < 0.001), while irisin (40 nmol/L) treatment significantly reversed these reductions (*p* < 0.01). As shown in Figure 5A, knockdown of AMPK by siRNA significantly attenuated the protective effect of irisin. For NF-κBp65, overexpression of NF-κBp65 was observed in 5 *μ*g/ml LPS-treated cells (*p* < 0.001, Figure 5B). However, 40 nmol/L irisin treatment greatly inhibited the expressions of NF-κBp65 (*p* < 0.001). Interestingly, AMPK siRNA-pretreatment abolished irisin’s inhibition effect on NF-κBp65 expression. Apart from NF-κBp65, pro-inflammatory cytokines (IL-1β, IL-6, TNF-α) were also dramatically increased after 5 *μ*g/ml LPS administration (*p* < 0.001 for IL-6, IL-1β and TNF-α, Table 3). These increases were inhibited by irisin (40 nmol/L) pretreatment. Furthermore, 5 *μ*g/ml LPS induced overexpression of iNOS and COX-2 in PC12 cells (*p* < 0.001 for iNOS and COX-2, Figures 5C,D). Irisin treatment reversed these abnormal expressions. However, AMPK siRNA-pretreatment abolished irisin’s function.

**TABLE 3:** Effects of irisin on IL-1β, IL-6 and TNF-α expressions in PC12 cells.

Group	Irisin dose (nmol/L)	IL-1β (pg/ml)	IL-6 (pg/ml)	TNF-α (pg/ml)
Control	0	12.4 ± 3.1	35.1 ± 9.2	21.4 ± 6.5
LPS	0	42.1 ± 3.7^***^	264.1 ± 12.1^***^	135.2 ± 8.3^***^
Irisin + LPS	10	36.2 ± 3.2	224.0 ± 13.2	124.5 ± 7.8
	20	31.9 ± 4.4	187.2 ± 12.1^##^	98.7 ± 7.9^#^
	40	23.5 ± 3.6^##^	137.2 ± 14.4^###^	78.5 ± 6.8^##^
SiRNA + irisin + LPS	40	37.4 ± 3.0^$^	201.4 ± 12.1^$$^	106.6 ± 8.7^$^

LPS, lipopolysaccharide. Values were expressed as mean ± SEM. Each group contains six rats. Data analysis was performed using Dunnett’s t-test. ^***^ *p* < 0.001 vs. control group; ^#^ *p* < 0.05, ^##^ *p* < 0.01 and ^###^ *p* < 0.001 vs. LPS group; ^$^ *p* < 0.05 and ^$$^ *p* < 0.01 vs. LPS + 40 nmol/L irisin group.

### Effects of Irisin on Lipopolysaccharide-Induced Oxidative Stress

**FIGURE 6:**
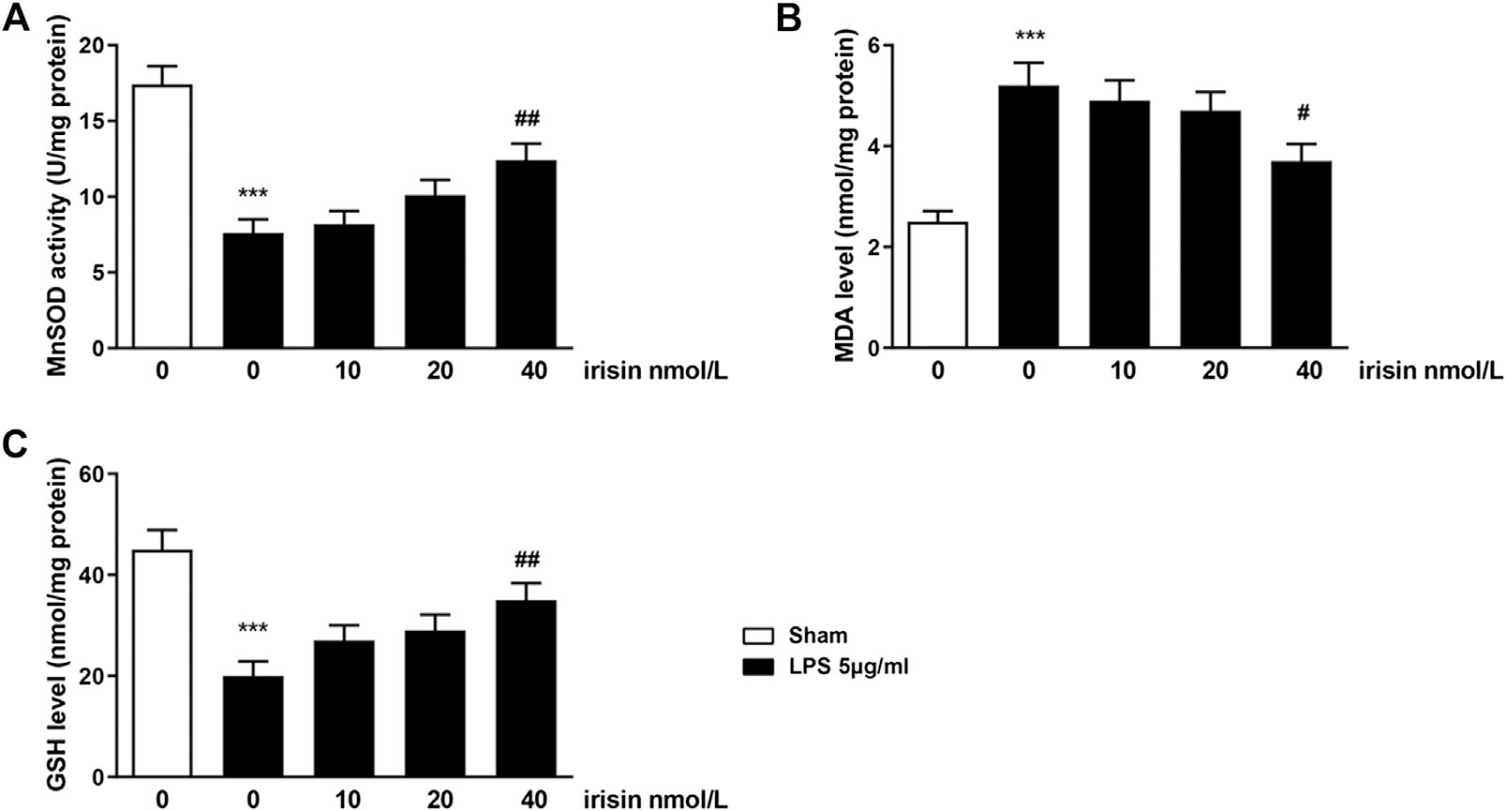
Effects of irisin on Mn-superoxide dismutase (MnSOD) activity **(A)**, malondialdehyde (MDA) level **(B)** and glutathione level **(C)** in LPS-challenged PC12 cells (***p = 0.000066 and ^##^ *p* = 0.0073 for MnSOD activity; ***p = 0.00028 and ^#^ *p* = 0.023 for MDA level; ***p = 0.00043 and ^##^ *p* = 0.0072 for GSH level). Data are presented as Mean ± SEM, n = 5. ***p < 0.001 when compared with control group; ^#^ *p* < 0.05 and ^##^ *p* < 0.01 when compared with LPS group.

To preliminary explore whether irisin’s protective function on nerve cells was correlated with oxidative stress, the expressions of oxidative-stress markers in PC 12 cells including GSH, MDA and MnSOD were evaluated. The results showed that the expressions of GSH and MnSOD were decreased and the expression of MDA was increased after LPS administration (*p* < 0.001, Figure 6), and irisin treatment reversed the above results (*p* < 0.01 for GSH and MnSOD, *p* < 0.05 for MDA).

## Discussion

When spinal cord is lacerated by a sharp penetrating force, contused by a blunt force, or infarcted by a vascular insult that usually occurs in traffic accident or sport, devastating neurological deficits begin because of initial mechanical injury including immediate hemorrhage and ischemia, and a long period of secondary damages including oxidative stress, inflammation, necrosis and apoptosis (Silva et al., 2014). Understanding the physiological mechanism of secondary and chronic phase of SCI is of vital importance, for it is beneficial to exploring promising therapies and minimizing the extension of the lesion. Our study suggested that irisin possesses neuroprotective effect against motor dysfunction in SCI rats. This positive function was associated with irisin’s modulation of inflammatory and neurotoxic mediators (IL-1β, IL-6, TNF-α, COX-2 and iNOS) via AMPK-NF-κB pathway.

The human spinal cord is protected by the vertebral column, and composed of gray matter located centrally and white matter marginally. The gray matter is made up of interneurons, cell bodies and dendrites of efferent neurons, the entering fibers of afferent neurons, and glial cells. The groups of myelinated axons mainly in white matter are connection of the spinal nerve and the brain (Silva et al., 2014). SCI often leads to the impairment of motor function, which is directed by the brain through spinal nerve. In our study, were used rats to establish a compression SCI model because rats share similar spinal anatomy and pathological features with humans. After being compressed the T9 spinal cord with a vascular clip, rats’ motor function was greatly inhibited, as observed on day 7 in BBB scoring test and inclined plane test. The motor function of SCI rats seems to gradually recover over the time. Results of HE staining showed that the gray matter and white matter were not normally arranged, with the neurons in the anterior horn shrunken and pale homogenous cytoplasm. Significant decrease of Nissl bodies in the SCI group also revealed the pathology changes in spinal cord tissue. The spinal cord neuron damage in SCI rats was observed more clearly by ultrastructural analysis. The afore-mentioned results indicated that SCI model of rat in our study, which had been confirmed by previous studies (Zhang et al., 2013; Zhao et al., 2016), was successfully established.

In our study, irisin levels in the plasma and spinal cord tissue on day 28 were found to significantly decline in SCI rats compared with those in the sham rats. A previous study conducted by Albayrak et al. showed that irisin levels increased in plasma, brain and spinal cord tissues with SCI, which was inconsistent with our results (Albayrak et al., 2015). Here we provide the following explanation. In Albayrak et al.’s study, the irisin levels in plasma and tissues were detected 24h post-surgery, while irisin levels in plasma and spinal cord tissue in our study were tested 28 days after SCI surgery. As is known, irisin is a metabolic hormone secreted mainly in skeletal muscle, it can also be produced in other tissues including plasma, brain, liver and kidney, and released into circulation (Aydin, 2014). At the early stage of SCI, irisin level rose because of the demand of energy consumption. In our study, however, irisin level was low in SCI rats compared with that in sham rats, which may be due to the reason that SCI rats with motor dysfunction were almost unable to move and the exercise of sham rats was much more than that of SCI rats, given that irisin level is positively correlated with exercise (Daskalopoulou et al., 2014; Huh et al., 2014). However, further investigations are warranted to verify this hypothesis. After exogenous irisin treatment, especially at 10 *μ*g/kg, the irisin levels in SCI rats were significantly improved. From the results of behavior evaluation tests and histomorphology tests, we could see that exogenous irisin exerted neuroprotective effect on spinal cord. The protective effect of 10 *μ*g/kg irisin is better than that of 5 *μ*g/kg irisin, and the recovery of motor function improved significantly after repeated irisin administration. It appears irisin exerted its neuroprotective function directly on the spinal cord because the variation trends of irisin levels in different groups in serum and spinal cord were consistent.

Inflammatory factors including IL-1β, IL-6 and TNF-αand neurotoxic mediators including COX-2 and iNOS are regulated by NF-κB (Jiang et al., 2017). They are associated with a variety of diseases like cancer, atherosclerosis, kidney diseases and myocardial infarction (Askari et al., 2018), and also play vital roles in evolution of secondary injury of SCI (Conti et al., 2003; Hohlfeld et al., 2007; Silva et al., 2014; Cao et al., 2015). In our study, the expression levels of these cytokines were significantly increased after surgery. In contrast, exogenous irisin treatment decreased all these cytokines in the spinal cord, and the variation trends of these cytokines in different groups were the same as the results obtained from behavior tests, indicating that irisin exert its neuroprotective effect by suppressing these factors. The correlation between irisin and these cytokines in neurons was also presented by other investigators (Peng et al., 2017).

AMPK is a metabolic kinase, which not only regulates the maintenance of energy metabolism but also involves in the regulation of many other physiological equilibria via an integrated signaling network (Salminen and Kaarniranta, 2012). It is demonstrated that NF-κB is a key element involved in AMPK signaling (Russo et al., 2014). Our research found that SCI surgery induced decreased expression of pAMPK in the spinal cord, which is in line with the previous reports (Meng et al., 2018; Lin et al., 2019). Whereas, the decreases were reversed by chronic irisin treatment. In addition, activated NF-κB was inhibited by chronic irisin treatment in SCI rat, indicating that irisin’s positive effects on SCI was related to the inhibition of NF-κB expression. Interestingly, when Compound C (AMPK inhibitor) was applied, AMPK was suppressed, and NF-κB expression, as well as its downstream factors including IL-1β, IL-6, TNF-α, COX-2 and iNOS, were significantly improved. These data implied that the inhibitory effect of irisin on IL-1β, IL-6, TNF-α, COX-2 and iNOS was achieved by controlling the AMPK-NF-κB pathway. Similar mechanism of irisin’s positive function was observed in Zhang et al.’s study that irisin activated AMPK and inhibited phosphorylation of NF-κB p65 while decreasing the expression of pro-inflammatory genes in rat INS-1E cells under glucolipotoxic conditions (Zhang et al., 2018). The mechanism of irisin’s neuroprotective effect was further confirmed in our *in vitro* experiment, that irisin reduced the expressions of IL-1β, IL-6, TNF-α, COX-2 and iNOS in cultured neurocytes challenged to LPS and elevated the cell survival rate, whereas these effects of irisin was absent in AMPK-silenced condition. In summary, our study is the first to report the chronic treatment effect of irisin on spinal cord injury, differing from Albayrak et al.’s study that evaluated the effect of irisin on acute spinal cord injury (Albayrak et al., 2015). The protection against SCI of irisin was strongly associated with the AMPK- NF-κB pathway. In our exploratory experiment regarding oxidative stress, it can be seen that irisin can regulate the oxidative stress-related markers including GSH, MDA and MnSOD, suggesting irisin exerted its function also via suppressing oxidative stress. The detail mechanism should be determined by further study.

## Conclusion

The present study demonstrated that irisin can ameliorate the motor dysfunction induced by SCI surgery in rats, indicating irisin is a potential candidate for clinical SCI therapy. The underlying mechanism of this effect partly depends on reducing the levels of IL-1β, IL-6, TNF-α iNOS and COX-2 via AMPK-NF-κB signaling pathway.

## Data Availability Statement

The raw data supporting the conclusions of this manuscript will be made available by the authors, without undue reservation, to any qualified researcher.

## Ethics Statement

The animal study was reviewed and approved by Animal Care and Use Committee of Zhejiang Pharmaceutical College.

## Author Contribution

XY and XJ designed the study, ZS, JC, CW, ZG, SY, LC, LX, ZC and WN conducted the experiment and analyzed the data, ZS wrote the manuscript draft and XY made the revision. All authors read and approved the final manuscript.

## Funding

This work was funded by Zhejiang Province Public Welfare Technology Application Research Project of China (LGF18H090014), Public science and technology project of Ningbo (202002N3151) for XY. Science and Technology Project of Zhejiang Medical and Health Department (2020KY289), Ningbo Natural Science Foundation (2019A610300), the Science and Technology Project of Yinzhou, Ningbo for XJ. Ningbo Natural Science Foundation (2019A610320), Science and technology plan of Zhejiang University Student (2018R458002) for LC. Zhejiang Provincial Natural Science Foundation (Q20H010022), Ningbo Natural Science Foundation (2019A610301) for LX. Zhejiang Province Public Welfare Technology Application Research Project of China (LGF18H090029) for ZC. Scientific Research Fund of Zhejiang Provincial Education Department (Y201840551) for WN. Ningbo Natural Science Foundation (2019A610218) for ZS.

## Conflict of Interest

The authors declare that the research was conducted in the absence of any commercial or financial relationships that could be construed as a potential conflict of interest.
